# Clinical characteristics, allergic response to autologous semen, and desensitization in patients with postorgasmic illness syndrome

**DOI:** 10.1093/sexmed/qfad068

**Published:** 2024-01-17

**Authors:** Guang-Peng Xi, Ruo-Xuan Yang, Jing Zhang, Yue-Mei Ma, Xiao-Yan Zhong

**Affiliations:** Department of Allergy, The Second Affiliated Hospital of Harbin Medical University, Harbin, 150081, China; Department of Pharmacology, College of Pharmacy, Harbin Medical University, Harbin, 157131, China; Department of Allergy, The Second Affiliated Hospital of Harbin Medical University, Harbin, 150081, China; Department of Allergy, The Second Affiliated Hospital of Harbin Medical University, Harbin, 150081, China; Department of Microbiology, College of Basic Medicine, Harbin Medical University, Harbin, 157131, China

**Keywords:** postorgasmic illness syndrome, clinical characteristics, seminal fluid, autologous immunoreactions, desensitization therapy

## Abstract

**Introduction:**

Postorgasmic illness syndrome (POIS) is rare and includes a cluster of physical and cognitive symptoms that occur after ejaculation. The pathogenesis and effective treatments remain unclear.

**Aim:**

This study aimed to characterize the symptomatology of POIS, study the allergic response of autologous semen in patients and controls, and evaluate the effects of desensitization therapy.

**Methods:**

The clinical characteristics of 24 Chinese patients with POIS were analyzed. Skin prick tests, intracutaneous tests, and specific IgE detection were performed with autologous semen. Five patients were desensitized via subcutaneous injections of autologous semen.

**Outcomes:**

Evaluated outcomes included the clinical features of POIS; scores of the Self-rating Anxiety Scale (SAS), Self-rating Depression Scale (SDS), and visual analog scale (VAS) of symptoms; skin reactions; desensitization with diluted autologous seminal fluid; and the IgE reactivity patterns of immunoblotting and enzyme-linked immunosorbent assay in vitro.

**Results:**

The most common symptom cluster was the general cluster, and the most prevalent symptoms were extreme fatigue and inattention. A total of 66.67% (14/21) of the patients had no symptoms or milder symptoms after nocturnal emission than after intercourse or masturbation. Of the patients, 87.5% (21/24) had psychiatric symptoms and 53.85% (7/13) had abnormal sex hormone levels. The SAS and SDS scores of the high and low VAS groups were significantly higher than those of the control group. Pearson analysis showed that the correlation coefficient between the SAS and VAS was 0.607 (*P* < .01) and that between the SDS and VAS was 0.490 (*P* < .05). The patients and healthy donors all had positive intracutaneous test results with their own semen, negative skin prick test results, and no IgE specific to autologous semen. Most patients (4/5) did not achieve ideal therapeutic effects with desensitization.

**Clinical Implications:**

Allergy is not the main pathogenesis of POIS, and desensitization with autologous semen is not effective for most patients.

**Strengths and Limitations:**

This project included the largest number of patients with POIS in China and assessed the allergic response to autologous semen and the effect of desensitization therapy. There is no objective method for evaluating the efficacy of desensitization with autologous semen.

**Conclusions:**

IgE-mediated semen allergy is not the main pathogenesis of POIS, and there is a positive chance that POIS is related to psychological factors. Most patients do not respond to desensitization with autologous semen, and POIS treatment should be individualized, especially in cases with uncertain causes.

## Introduction

Postorgasmic illness syndrome (POIS), a rare disorder recognized by the National Institutes of Health of the United States, includes a cluster of physical and cognitive symptoms that occur after ejaculation. It was first reported by Waldinger and Schweitzer^1^ in 2002; later, Waldinger et al proposed 5 preliminary diagnostic criteria for POIS,^2^ which were recently adapted by Strashny.^3^ Over 70 confirmed cases of POIS have been cited in the medical literature^4^; however, the number of self-reported cases in online forums for the disease has increased rapidly. Owing to the lack of awareness of the syndrome and the limited number of studies, many cases of POIS may go undiagnosed.

The pathogenesis of POIS remains unclear. Ashby and Goldmeier^5^ proposed that a disordered cytokine or neuroendocrine response causes symptoms of POIS. Dexter^6^ postulated that POIS could be caused by a lack of progesterone, and this view was supported by a report of successful treatment with human chorionic gonadotropin for a patient with testosterone deficiency,^7^ although only some patients have disturbed hormone levels. Pierce et al^8^ hypothesized that POIS was associated with sympathetic dysregulation, and Waldinger et al^2^ showed that 88% of affected patients had a positive skin test result for autologous semen and concluded that type I and IV allergies to their own semen may contribute to symptoms of the illness. However, no healthy controls were included in the trial, and semen-specific IgE was not detected in the sera of the patients. Later, a case report^9^ from South Korea noted the presence of semen-specific IgE in patients with POIS and healthy controls. Yet, a case report from Depreux et al^10^ did not reveal an IgE-mediated cause. Our previous research^11^ also showed that IgE-mediated semen allergy in men may not be the mechanism of POIS, and it suggested that patients with POIS may have a disorder of the endogenous μ-opioid receptor system. Similarly, a recent study^12^ hypothesized that repetitive eccentric contractions of the bulbospongiosus and ischiocavernosus muscles can lead to acute compression proprioceptive axonopathy and acute stress injury, which can disrupt the opioid-induced reward system and lead to POIS.

In 2015, we reported the first case of POIS in China.^11^ Since then, more patients suspected of having POIS have visited our clinic from all over China. In this study, we analyzed the clinical characteristics of 24 patients who met the diagnostic criteria for POIS,^2^^,^^3^ quantified the severity of each symptom, and assessed patients and healthy controls for anxiety and depression. Further studies were conducted in some patients and healthy volunteers, mainly to observe the allergic reactions to autologous semen via skin and serologic tests.

As the pathogenesis of POIS is unknown, no effective treatment has been established. Treatment with antihistamines and prednisone has been shown to be ineffective in managing symptoms, and treatment with benzodiazepines and selective serotonin reuptake inhibitors improves mood without affecting somatic symptoms.^1^ Flutamide treatment reduces libido and ejaculation frequency but has no effect on physical or psychological symptoms.^1^ By inhibiting ejaculation, the highly selective α1A-blocker silodosin was effective in 57% of patients,^13^ but the lack of ejaculation was not accepted by many patients, especially those preparing for pregnancy. In a patient with POIS with left epididymitis, POIS symptoms—especially rash and headache—improved after bilateral epididymectomy and bilateral vasoligation.^14^ Moreover, some cases^9^^,^^15^^,^^16^ reported that desensitization therapy with autologous semen has a certain therapeutic effect. In this study, 5 patients voluntarily requested desensitization with autologous semen. The treatment process and results are presented here.

This study aimed to characterize the symptomatology of POIS, study the allergic response of autologous semen in patients and controls, and evaluate the effects of desensitization therapy.

## Methods

### Study participants

Twenty-four outpatients suspected of having POIS were enrolled between April 2020 and October 2023, who fulfilled at least 4 of the 5 diagnostic criteria of POIS^2^^,^^3^:

- One or more symptoms from among these clusters:
  - *General:* extreme fatigue/exhausted, palpitations, problems finding words/dysarthria, concentration problems, quickly irritated, photophobia, and depressed mood
  - *Flu-like:* feverish/extreme warmth, desudation, and shivering/feeling cold
  - *Head:* headache and heavy and foggy feelings in the head
  - *Eyes:* conjunctival congestion, blurred vision, itching/watery eyes, dry eyes, and painful eyes/pressure on the eyes
  - *Nose:* congested nose, sneezing, and runny nose
  - *Throat:* dry mouth, thirst, tickling cough, hoarse voice, and sore throat
  - *Muscle:* muscle tension in the back or neck and muscle weakness, pain, and stiffness
- Symptoms begin immediately or within a few hours after ejaculation that is initiated by sex, masturbation, or nocturnal emission.
- Symptoms occur after all or almost all ejaculations or in at least 1 ejaculatory setting (sex, masturbation, or nocturnal emission).
- Most symptoms last for about 2 to 7 days.
- Symptoms disappear spontaneously.

Patients who had not been actively recruited were interviewed and asked about their complaints (main symptoms, age at onset, duration, clinical course, and ejaculatory settings), medical history, existing allergies, possible psychological or psychiatric disorders, and sexual function. Premature ejaculation (PE) was defined as an intravaginal ejaculation latency time <60 seconds and was determined according to its estimation. Six of the 24 patients with suspected POIS (P1-P6) and 6 healthy controls (C1-C6) agreed to participate in skin and serologic tests of autologous semen, and 5 patients (P1-P3, P5, P6) voluntarily requested desensitization with autologous semen. The healthy volunteers were patients who had come in for a physical examination. The inclusion criteria were as follows: no history of allergic disease, negative test results for common inhaled and ingested allergens and total IgE, not meeting the diagnostic criteria for POIS, and having no symptoms associated with POIS. This study was approved by the Ethics Committee of The Second Affiliated Hospital of Harbin Medical University. Informed consent for study participation was obtained from all participants.

### Visual analog scale, Self-rating Anxiety Scale, and Self-rating Depression Scale

The visual analog scale (VAS) ranged from 0 to 10 cm (0 = no complaints, 10 = worst attack) and consisted of 30 POIS-related symptoms: it was developed and used as a reference to the multi-VAS for rhinitis,^17^ and it quantified the severity of each symptom in 19 patients. According to the total VAS score, patients were divided into 2 groups with a dividing line of 100—namely, the group with high VAS scores (≥100) and the group with low VAS scores (<100). The Self-rating Anxiety Scale (SAS) and Self-rating Depression Scale (SDS), developed by Zung,^18^^,^^19^ were performed among the 19 patients and healthy controls. Differences in SAS and SDS scores among the high VAS, low VAS, and healthy control groups were assessed, as were the correlations between the SAS or SDS score and VAS score.

### Semen allergen extract

Fresh seminal fluid was collected in sterile centrifuge tubes by masturbation and allowed to liquefy undisturbed for 30 minutes at room temperature. The ejaculate was then diluted with 0.9% saline to a concentration of 1:10 000, 1:1000, 1:100, and 1:10 for skin testing. The remaining samples were stored at −80 °C until use.

### Skin tests

Six patients and 6 healthy volunteers underwent intracutaneous tests (ICTs) and skin prick tests (SPTs) with diluted seminal fluid according to a standard protocol.^20^ The positive and negative controls consisted of histamine (0.1 mg/mL) and 0.9% saline solution, respectively. Skin reactions to autologous semen were interpreted 15 minutes after the tests and found to be positive when the diameter of the wheal was ≥5 mm; a wheal <5 mm was regarded as a negative result. The skin test results were photographed indoors under fluorescent lighting at room temperature of approximately 24 °C.

### SDS-PAGE and Western blotting

Semen extracts from the affected patients and healthy controls were analyzed with SDS-PAGE (sodium dodecyl sulfate–polyacrylamide gel electrophoresis). Fifteen micrograms of protein were applied per lane. After electrophoresis, the gels were stained with 0.1% Coomassie Brilliant Blue R-250. Proteins were transferred to a polyvinylidene difluoride membrane. The membrane was saturated with 5% skim milk in 0.5% Tween-20/phosphate-buffered saline (PBST) for 2 hours at room temperature and incubated with the serum from the patients and controls at a dilution of 1:5 overnight at 4 °C. The strips were washed with PBST and incubated for 2 hours at room temperature with a 1:1000 dilution of horseradish peroxidase–conjugated anti-human IgE (Abcam). IgE-binding proteins were detected with an enhanced chemiluminescence substrate (Meilunbio) according to the manufacturer’s instructions.

### Enzyme-linked immunosorbent assay

Specific IgE levels in autologous semen were investigated with enzyme-linked immunosorbent assay (ELISA). Experimental wells of a 96-well plate were coated with 10 μg of seminal fluid diluted in carbonate buffer (pH, 9.6), incubated overnight at 4 °C, washed 3 times with PBST, and blocked for 2 hours with 1% bovine serum albumin at 37 °C. Autologous serum samples diluted 1:3 with blocking buffer were added, incubated overnight at 4 °C, washed, and incubated with 100 μL of anti-human IgE–horseradish peroxidase conjugate (Abcam). The assay was developed with a substrate solution (PNTK) in the dark, and the enzymatic reaction was stopped after 30 minutes of substrate incubation by the addition of 1-mol/L hydrochloric acid. The absorbance was measured at 450 nm with a spectrophotometer (Spectra MAX; Molecular Devices). Blank wells were coated with carbonate buffer without seminal fluid, and the other procedures were consistent with the respective experimental wells. All determinations were performed in triplicate.

### Desensitization therapy

Desensitization with autologous semen was done for 5 patients (P1-P3, P5, P6). The ejaculate was diluted with 0.9% saline to concentrations of 1:100 000, 1:10 000, 1:1000, 1:100, and 1:10. Desensitization treatments were performed by subcutaneous injection at the lateral upper arm, and diluent seminal fluid (0.2, 0.4, 0.6, 0.8, 1.0 mL) from concentrations of 1:100 000 to 1:10 was gradually injected once a week. We used 1.0-mL diluent of 1:10 concentration as the maintenance dose. The effects of the therapy were evaluated before and each month after the first injection. The patients were asked to score the severity of each POIS-related symptom in the last 2 weeks using the VAS and to describe each duration.

### Data record and analysis

Quantitative variables are described with mean and SD or median and range. Categorical variables (symptoms, complaints, and comorbidities) are summarized as numbers and percentages. Analysis of variance was used to assess the differences in SAS and SDS scores among the high VAS, low VAS, and healthy control groups. Pearson correlation analysis was used to evaluate the correlation between SDS/SAS and VAS in patients. *P* < .05 was regarded as statistically significant.

## Results

### Disease presentation

Patient characteristics are described in Table 1. The mean ± SD age of the 24 patients with suspected POIS was 24.17 ± 6.51 years. The average age of syndrome onset was 16.50 ± 4.19 years. The duration of symptoms was 7.11 ± 6.23 years, and complaints per episode lasted for 5.38 ± 2.10 days. The percentage of patients who developed symptoms within 30 or 60 minutes after ejaculation was 66.67% (16/24) and 83.33% (20/24), respectively. Of the patients, 66.67% (14/21) had no symptoms or milder symptoms after nocturnal emission than after intercourse or masturbation, and 87.50% (21/24) had psychiatric symptoms (anxiety, depression, obsessive compulsive disorder). Of the 13 patients, 7 (53.85%) had abnormal sex hormone levels: 4 had high progesterone levels, 2 had elevated prolactin levels, 2 had low testosterone levels, and 1 had reduced estradiol levels. Of the patients, 47.83% (11/23) had atopic constitution and 47.39% (9/19) had PE. The average age of the 6 healthy volunteers was 24 ± 2.19 years.

**Table 1:** Characteristics of 24 patients with POIS.

**Item**	**Mean ± SD (range) or No. (%)**
Age, y	
At suspected POIS	24.17 ± 6.51 (17-40)
At onset	16.50 ± 4.19 (11-30)
Duration of	
POIS, y	7.11 ± 6.23 (1-23)
Complaints per episode, d	5.38 ± 2.10 (1-8)
Symptoms occurred	
Within 30 min of ejaculation	16/24 (66.67)
Within 60 min of ejaculation	20/24 (83.33)
No/milder symptoms after nocturnal emission vs after intercourse or masturbation	14/21 (66.67)
Psychiatric symptoms	21/24 (87.50)
Abnormal sex hormone levels	7/13 (53.85)
Atopic males	11/23 (47.83)
Premature ejaculation	9/19 (47.39)

Abbreviation: POIS, postorgasmic illness syndrome.

### Symptomatology and symptom clusters

The most common symptom cluster was the general cluster (100%), followed by the nose (91.7%), head (87.5%), flu-like (75.0%), pharyngeal (66.7%), eye (62.5%), and muscular clusters (62.5%). Extreme fatigue and inattention were the most frequent symptoms, (92%) followed by feelings of loss (88%) and brain fog/confusion (88%). Conjunctival hyperemia (8%), hoarseness (13%), and photophobia (13%) were less common. The worst symptoms were extreme fatigue (total VAS score, 138), followed by brain fog (123), inattention (108), feelings of loss (102), and congestion of the nose (89). In addition to these common symptoms, 3 patients had symptoms of insomnia; 2 had cognitive disorder; and 1 each had overeating, hair loss, and erythema on the face. The frequencies of symptom clusters and individual symptoms are presented in Tables 2 and 3.

**Table 2:** Number of respondents grouped into each symptom cluster in 24 patients.

**Cluster**	**No.**	**%**
General	24	100.0
Flu-like	18	75.0
Head	21	87.5
Eyes	15	62.5
Nose	22	91.7
Throat	16	66.7
Muscle	15	62.5

**Table 3:** Number of responses in 24 patients and total VAS scores of 19 patients exhibiting specific symptoms.

**Cluster: complaint**	**No.**	**%**	**Total VAS of 19 patients**
General			
Extreme fatigue/exhausted	22	92	138
Palpitations	12	50	52
Problems finding words/dysarthria	12	50	68
Difficult to concentrate	22	92	108
Irritability	14	58	50
Photophobia	3	13	23
Depressed mood	21	88	102
Flu-like			
Feverish/extreme warmth	13	54	34
Desudation	16	67	69
Shivery/feeling cold	6	25	28
Head			
Headache	14	58	73
Pressure or heavy	12	50	83
Foggy	21	88	123
Eyes			
Conjunctival congestion	2	8	34
Blurred vision	6	25	32
Itching/watery eyes	5	21	34
Eye drying	8	33	45
Painful eyes/pressure on eyes	6	25	40
Nose			
Congestion	15	63	89
Sneezing	12	50	45
Watery/runny	14	58	66
Throat			
Dry mouth	11	46	45
Thirst	9	38	45
Cough	4	17	17
Hoarse voice	3	13	17
Sore	6	25	27
Muscle			
Tension	5	21	33
Weakness	11	46	71
Pain	5	21	37
Muscle stiffness	5	21	35
Particular			
Insomnia	3	13	15
Cognitive disorder	2	8	15
Overeating	1	4	4
Hair loss	1	4	5
Erythra on the face	1	4	3

Abbreviation: VAS, visual analog scale.

### VAS, SAS, and SDS

**Figure 1:**
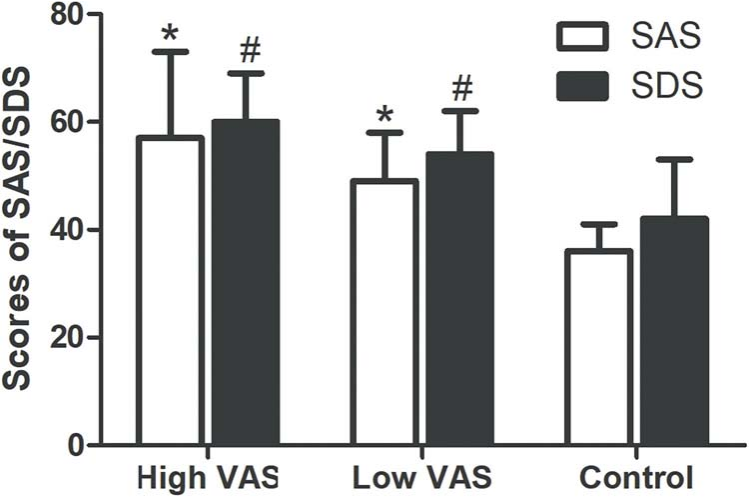
The differences in SAS and SDS scores were compared among the high VAS, low VAS, and healthy control groups. ^*^P < .05: the SAS score of the high or low VAS group vs the control group. ^#^*P* < .05: the SDS score of the high or low VAS group vs the control group. SAS, Self-rating Anxiety Scale; SDS, Self-rating Depression Scale; VAS, visual analog scale.

There were 7 patients in the high VAS group; the mean ± SD VAS, SAS, and SDS scores were 136 ± 36, 57 ± 16, and 60 ± 9, respectively. There were 12 patients in the low VAS group; the average VAS, SAS, and SDS scores were 69 ± 23, 49 ± 9, and 54 ± 8. The average SAS and SDS scores in healthy control were 36 ± 5 and 42 ± 11. The VAS, SAS, and SDS scores for each participant are displayed in Tables 3 and 4. The SAS and SDS scores of the high and low VAS groups were both significantly higher than those of the control group (Figure 1A; *P* < .05). Pearson analysis revealed that the correlation coefficient between the SAS and VAS was 0.607 (*P* < .01) and that between SDS and VAS was 0.490 (*P* < .05).

**Table 4:** VAS, SAS, and SDS scores of patients with postorgasmic illness syndrome and controls.

	**Score**		
**Participants**	**VAS**	**SAS**	**SDS**
P	192	87	73
P	184	51	52
P	122	70	65
P	117	57	60
P	115	52	65
P	113	41	52
P	110	43	50
P	98	56	58
P	98	51	53
P	91	60	61
P	87	51	60
P	82	56	60
P	73	46	52
P	68	46	60
P	53	40	51
P	52	53	47
P	49	35	58
P	41	32	32
P	33	56	57
C	—	42	37
C	—	41	40
C	—	33	28
C	—	32	40
C	—	36	62
C	—	31	43

Abbreviations: C, health control; P, patient; SAS, Self-rating Anxiety Scale; SDS, Self-rating Depression Scale; VAS, visual analog scale.

### Skin testing

**Figure 2:**
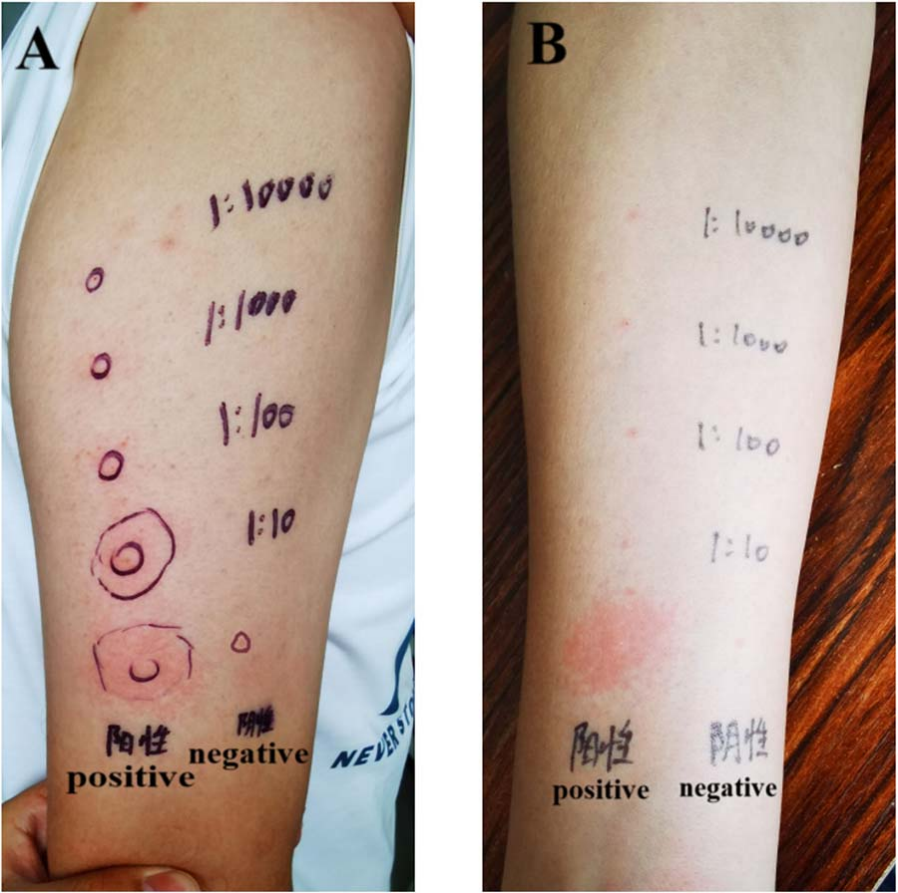
Photographs of (A) ICTs and (B) SPTs of 1 patient. *Positive* and *negative* represent positive and negative control, respectively. The photos were taken indoors under fluorescent lighting at room temperature of about 24 °C.

Six patients and 6 healthy controls received ICTs and SPTs with autologous semen. The results are summarized in Table 5. In the ICTs, 1 patient (P1) tested negative (3-mm wheal) at a 1:100 dilution, and all other patients and controls tested positive (≥5 mm) at 1:10 and 1:100 dilutions. The SPT results were negative in all patients and controls at every dilution. Most participants’ wind clusters were reddish. Photographs of ICTs and SPTs of 1 patient (P5 as an example) are presented in Figure 2.

**Table 5:** Intracutaneous skin and skin prick tests.^a^

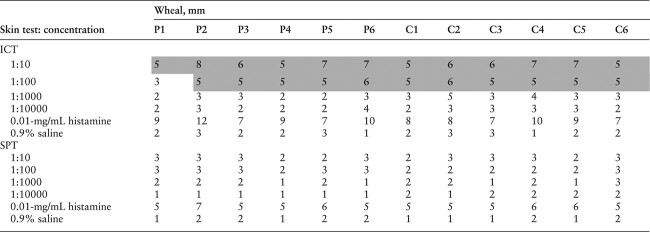												

Abbreviations: C, health control; ICT, intracutaneous test; P, patient; SPT, skin prick test.

^a^Results are expressed as wheals, as described in *Allergology*.^20^ Values in bold and dark backgrounds indicate positive results.

### SDS-PAGE and Western blotting

**Figure 3:**
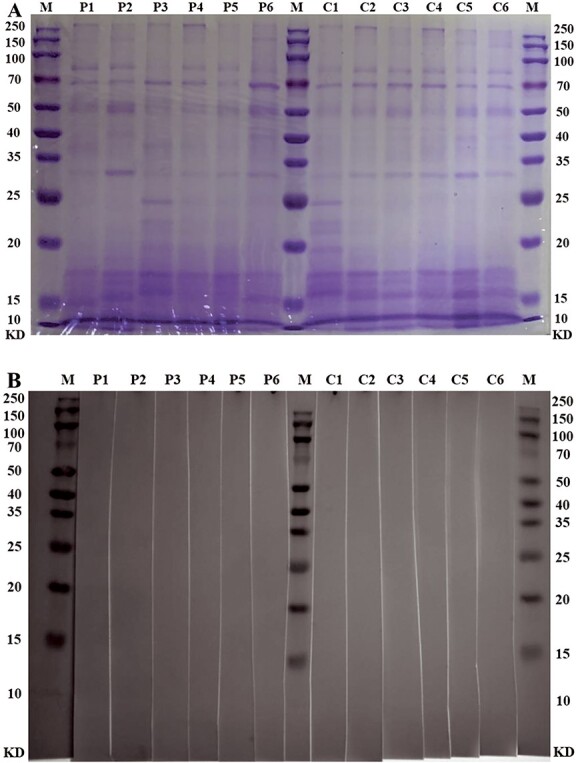
SDS-PAGE and IgE immunoblotting of the patients’ and healthy controls’ seminal fluid. (A) SDS-PAGE: lane M, molecular mass markers; lane P1-P6, affected patients’ seminal fluid; lane C1-C6, controls’ seminal fluid. (B) IgE immunoblotting: lane M, molecular mass markers; lane P1-P6, electrophoresis with patients’ seminal fluid and incubation with their own serum; lane C1-C6, electrophoresis with controls’ seminal fluid and incubation with their own serum. SDS-PAGE, sodium dodecyl sulfate–polyacrylamide gel electrophoresis.

SDS-PAGE of seminal fluid showed that the size and distribution range of the protein bands were similar between patients and controls (Figure 3A). IgE immunoblotting of autologous seminal fluid samples, incubated with the serum of the patients and controls, revealed no IgE-binding bands (Figure 3B).

### ELISA

**Figure 4:**
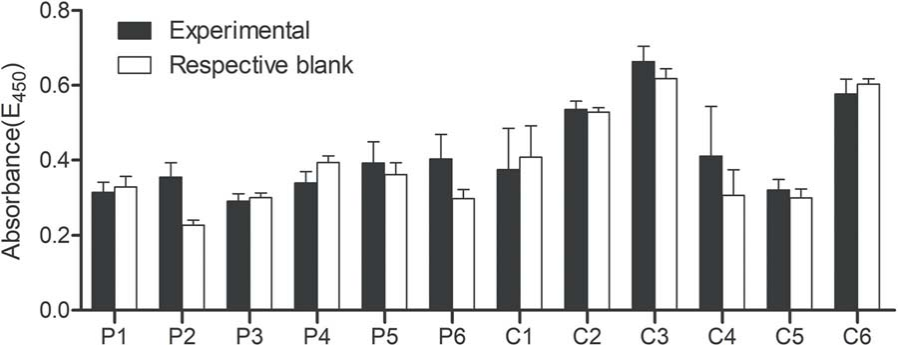
IgE reactivity of sera to autologous semen measured by enzyme-linked immunosorbent assay. C1-C6, healthy controls; P1-P6, affected patients.

Semen-specific IgE was not detected by ELISA in the patients or healthy controls (Figure 4). The absorbance values of the patients and controls were similar to that of the respective blank.

### Effect of desensitization therapy

Of the 5 patients (P1-P3, P5, P6) who were treated with desensitization therapy, 4 patients (P1, P3, P5, P6) did not experience the desired therapeutic effect. P6 underwent desensitization for 5 months, and the severity of symptoms decreased by approximately 15%; however, their duration progressed from 1 to 6 days. The other 3 patients (P1, P3, and P5) were desensitized for 7, 8, and 9 months, respectively. There was no marked improvement in 2 patients (P1 and P5), and 1 patient (P3) experienced worsening symptoms. Therefore, these 4 patients discontinued treatment. Yet, P2 demonstrated significant improvement: POIS symptoms disappeared by 60% after 8 months of desensitization. This patient had been desensitized for 20 months, experiencing only nasal congestion accompanied by slight dizziness, with no extreme fatigue or other symptoms, which did not affect normal life.

## Discussion

To date, approximately 70 confirmed cases of POIS have been reported,^4^ yet its pathogenesis and effective treatment have not yet been fully elucidated. In this study, we analyzed the clinical characteristics of 24 Chinese patients with POIS and performed skin and serologic tests with autologous semen in 6 patients and 6 healthy volunteers; desensitization therapy with autologous semen was performed among 5 patients.

Our study demonstrated that symptoms appeared within 30 or 60 minutes of ejaculation in 66.67% and 83.33% of patients, respectively. A previous study^2^ also found that symptoms started within 30 minutes after ejaculation in 87% of patients with POIS. Of the individual symptoms, extreme fatigue and inattention appeared most frequently, followed by depressed mood and brain fog/confusion; conjunctival hyperemia, hoarseness, and photophobia were less common. In a study by Waldinger et al,^2^ the most frequent complaints of patients were feverishness, extreme fatigue, concentration difficulties, and quick irritation, while photophobia, hoarse voice, and depressed mood were less common. Reisman reported^13^ that the most prevalent complaints were extreme fatigue, heavy head pressure, nose congestion, and muscle tension. These findings suggest that the clinical manifestation of POIS varies and extreme weakness is common in most patients. The worst symptoms were extreme fatigue, followed by brain fog, inattention, and feelings of loss. Conversely, this is related to the high frequency of these symptoms, and it reflects that these systemic or head symptoms have the greatest impact on patients’ normal lives. So, some patients will be unable to carry out normal work and study and will reject sex, leading to serious mental and psychosocial consequences.^21^ Atopic constitution was observed in 47.83% of patients in our study and 58% of patients in the Waldinger et al study. However, Reisman noted that of the 14 patients with POIS, only 4 had an allergy. Similarly, Natale et al^4^ stated that only 16% of patients suspected of POIS had allergic reactions. Therefore, it cannot be concluded that the majority of patients with POIS are allergic, and it cannot be inferred that allergy is the pathogenesis of POIS.

Similar to our previous study,^11^ the patients and healthy controls all had positive ICT results with their own semen, whereas negative SPT results and no specific IgE to autologous semen were detected in their serum. These results suggest that allergy to autologous semen is not responsible for the development of POIS. The positive results of ICTs were not achieved through the IgE pathway but may be due to the nonspecific effects of some components contained in the seminal fluid—such as monocyte chemotactic protein 1, ex-IL-8, C-X-C ligand 1/Th17,^22^ and high concentrations of spermine^23^—which could directly stimulate the skin and cause wheals when injected into the skin in large quantities.^23-25^

Of the patients, 66.67% had no symptoms or milder symptoms after nocturnal emission than after intercourse or masturbation. Previous studies^3^ showed that 36% of patients with suspected POIS did not always experience symptoms after nocturnal emissions. This difference may be an important clue to the etiology of POIS. Similar to a recent hypothesis,^12^ we speculated that during masturbation or intercourse, the bulbospongiosus and ischiocavernosus muscles that control penile erection and lengthening were repeatedly stimulated. Excessive eccentric contractions in the muscle spindles led to acute compression proprioceptive axonopathy and acute stress injury, resulting in autonomic nervous system confusion and spermidine consumption, which can destroy opioid-like reward systems and eventually cause POIS. In the process of nocturnal emission, the corpus cavernosum muscles were not intensely stimulated; therefore, there were no or only mild POIS symptoms. Of the patients, 47.39% had PE, similar to the 56%^2^ and 47%^4^ in previous studies; this demonstrates that a considerable number of patients with POIS have associated PE. This may be a result of the Piezo2 channels of the proprioceptive axon terminals of the bulbospongiosus and ischiocavernosus muscle spindles experiencing acute stress-induced mechanoenergetic lesion,^12^ as Piezo2 is involved in the pathogenesis of PE.^26^

In our study, 87.50% of the patients had psychiatric symptoms (anxiety, depression, obsessive compulsive disorder). Natale et al^4^ reported depression and generalized anxiety disorder in 45% of patients with suspected POIS. Our study found that the SAS and SDS scores of patients with POIS in the high and low VAS groups were significantly higher than those of healthy controls. The correlation analysis results revealed a significant positive correlation between SAS and SDS scores and VAS scores. This made us highly suspect that POIS may be related to psychological factors; however, it is unclear whether these psychiatric symptoms are complications of POIS or if they predate the onset of POIS symptoms.

Possibly, diagnoses other than POIS are involved. Postcoital dysphoria or postcoital psychological symptoms can include tearfulness and feelings of melancholy, depression, anxiety, agitation, or aggression, which can mimic the psychological burden of POIS; however, these symptoms often last only up to an hour after sexual intercourse.^27^ In some South Asian communities, it is believed that it takes 40 drops of blood to produce 1 drop of bone marrow and 40 drops of bone marrow to produce 1 drop of semen^28^; therefore, the loss of semen due to ejaculation can produce anxiety and neurosis. Moreover, it is worth noting that in a previous retrospective study^29^ of 4211 men attending andrology and sexual medicine clinics, 8.4% reported any sense of guilt after masturbation and had more psychiatric comorbidities than the remaining sample. For patients with POIS, especially young unmarried patients, we should pay attention to analyzing whether their symptoms are related to self-blame after masturbation.

In addition, 53.85% of patients had abnormal sex hormone levels. The types of abnormal hormones were not the same in these patients. Two existing studies with larger enrollments^2^^,^^13^ reported no abnormal changes in sex hormone levels in patients. However, some cases^7^^,^^9^^,^^30^ have reported abnormal changes in different kinds of sex hormones, such as estradiol, testosterone, prolactin, and free testosterone. Future studies with larger samples are needed to determine this regularity.

Few cases of desensitization to POIS with autologous semen have been reported. Wrotynska-Barczynska et al found^16^ that flu-like symptoms in a patient resolved after 1 month of desensitization, and most symptoms were in remission by 14 months. One case of intralymphatic desensitization^9^ showed some efficacy at 8 and 15 months. Waldinger et al reported^15^ that desensitization resulted in 60% and 90% amelioration of POIS complaints in 2 patients at 31 and 15 months, respectively. However, immunotherapy was administered to a Brazilian patient with POIS with minimal symptomatic improvement.^31^ In our study, most patients undergoing desensitization therapy did not achieve a therapeutic effect, and only 1 patient’s symptoms were effectively relieved. This may be due to insufficient treatment time (5-9 months); the effects of treatment may take more time to appear. It is more likely, though, that desensitization with autologous semen is not suitable for patients with POIS, given that we did not find specific IgE to autologous semen in our patients. It is uncertain whether the effectiveness of treatment in some patients is related to psychological reassurance or the nonspecific effects of repeated subcutaneous injections. Randomized double-blind controlled studies with larger samples are needed to confirm this.

In view of the diversity of clinical manifestations and the fact that no single theory could explain the pathogenesis of POIS, we suggest that treatment should be individualized. For example, patients with sex hormone disorders could be given corresponding hormone supplements; patients with mostly allergy-like symptoms (eg, nose symptoms or rash) and detectable semen-specific IgE can be desensitized; patients with epididymitis or other organic diseases of the reproductive system could consider appropriate surgical treatment; patients who are not preparing for pregnancy could consider treatment to reduce ejaculation; and patients with behavioral and cognitive symptoms (eg, irritability or brain fog) or autonomic nervous system disorders (eg, flu-like symptoms or palpitations) could consider using riluzole and spermidine before sex. In addition, patients with psychiatric symptoms should receive psychological counseling and psychotropic medication.

There were some limitations in this study. To date, there is no objective method for evaluating the efficacy of desensitization with autologous semen, which can be estimated only by the subjective description of patients.

In conclusion, patients with POIS and healthy individuals had positive skin reactions to autologous seminal fluid, and no semen-specific IgE was detected in men with POIS, suggesting that allergy is not the main pathogenesis of POIS. Most patients did not respond to desensitization using autologous semen. Therefore, excessive eccentric contraction of the cavernous muscle of the penis during sexual activity may cause POIS, and psychological factors also play a significant role in its pathogenesis. In cases in which the cause of POIS is currently uncertain, treatment should be individualized.
